# Factors associated with place of death in Addis Ababa, Ethiopia

**DOI:** 10.1186/1472-684X-12-14

**Published:** 2013-03-26

**Authors:** Aderaw Anteneh, Tekebash Araya, Awoke Misganaw

**Affiliations:** 1 Addis Ababa Mortality Surveillance Program, College of Health Sciences, Addis Ababa University, Addis Ababa, Ethiopia

**Keywords:** Place of death, Health facility, Deceased, Addis Ababa

## Abstract

**Background:**

Dying at home is highly prevalent in Africa partly due to lack of accessibility of modern health services. In turn, limited infrastructure and health care deliveries in Africa complicate access to health services. A weak infrastructure and limited health facilities with lower quality in Ethiopia resulted poor health service utilization and coverage, high morbidity and mortality rates. We examined whether people in Addis Ababa died in health facilities and investigated the basic factors associated with place of death.

**Methods:**

We used verbal autopsy data of 4,776 adults (age>14 years) for the years 2006–2010 from the Addis Ababa Mortality Surveillance Program (AAMSP). The main data source of AAMSP is the burial surveillance from all cemeteries in Addis Ababa. We provide descriptive statistics of place of adult deaths and discussed their covariates using multivariate analyses.

**Results:**

Only 28.7% died at health facilities, while the remaining died out of health facilities. There was an increase trend in the proportion of health facility deaths from 25.3% in 2006 to 32.5% in 2010. The risk of health facility death versus out of health facility deaths decreased with age. Compared with those who had no education educated people were more likely to die at health facilities. The chance of in health facility death was a little higher for females than males while religion, occupational status and ethnicity of the deceased had no any significance difference in place of death.

**Conclusion:**

Both demographic and social factors determine where adults will die in Addis Ababa, Ethiopia. The majority of people in Addis Ababa died out of health facilities. The health system should also give special attention to the emerging non communicable diseases like cancer for effective treatment of patients.

## Background

In developed countries dying has become increasingly hospitalized and medicalized and place of death has, for some time now, been an issue of interest to public health policy and to palliative care in particular [1,2]. In contrast, dying at home is highly prevalent in Africa partly due to lack of accessibility of modern health services [3]. In turn, limited infrastructure and health care deliveries in Africa complicate access to health services [4].

Ethiopia is a country of diverse cultures, traditions and histories with poor health outcomes even by sub-Saharan Africa’s standards [5]. Poverty, backwardness, malnutrition, limited access to health services and unbalanced population growth coupled with harmful traditional practices are blamed for the poor health outcome of the country [5,6]. Despite extensive poverty and limited resources, in the last decade Ethiopia has made impressive progress in improving its health care system. The improvement is especially on training of health extension workers, expansion of health centers and improved staffing, proper provision of equipment, essential medicines and other supplies that led to a marked increase in the uses of primary health care services [7].

Despite all the efforts made to improve health care services, a weak infrastructure and limited health facilities with lower quality in Ethiopia resulted poor health service utilization and coverage, high morbidity and mortality rates [8,9]. As a result, most patients die in their own homes under the care of their families without getting appropriate medication in health services [10]. In addition, affordability of medical services plays also a great role to utilize services. For example, in the urban setting of Ethiopia, it is common that sick people from richer households seek medical care more often and with a greater intensity than those from poor families [11].

In developed countries, considerable literature studying the place of death of patients, and predictive factors for place of death indicated that; place of death determined by multiple factors such as culture, social, economy, demography and type of disease. More specifically, individual characteristics like age, sex, marital status, educational status, occupational status; disease processes, cause of death, family and general practitioner's support, social and economic support and health care system structure determine place of death [12-15].

Although, understanding where people die and associated factors from African context is very important for health planners and decision makers, data on place of death and its determinants are lacking. Therefore, the aim of this paper is to examine place of death and associated socio-demographic factors of the deceased to assist health planners and decision makers to improve the quality of health services; particularly the palliative care in Addis Ababa, Ethiopia.

## Methods

This work is part of the Addis Ababa Mortality Surveillance Program (AAMSP). The main data source of AAMSP is the burial surveillance which was initiated in 2001 at all cemeteries of Addis Ababa [16]. The surveillance is conducted by cemetery clerks who are regularly trained about death registrations in training workshops. For all cemeteries there are 13 supervisors who assisted the cemetery clerks. Currently, a total of 89 cemeteries are under surveillance. The variables registered by the cemetery clerks includes the following: name of the deceased, date of burial, age, sex, birth region, marital status, ethnicity, religion and specific address (house number, village name and phone number) of the deceased. Overall 85,584 deaths were registered during the consecutive five years time (2006–2010) period.

After excluding decedents whose ages were below 14 years, addresses were incomplete and bodies that were found dead; for randomly 10% selected records of deceased from the ongoing surveillance of burials verbal autopsies (VA) were administered. Verbal autopsy is postmortem interview with next of kin or other caregivers of the deceased about the signs and symptoms during terminal illness, is an alternative method for estimating the distribution of causes of death in a population [17].

Among all records selected for verbal autopsy, about 83% were completed successfully. The rest were not completed for the main reasons either respondents refused for interview or the households were not found. Verbal autopsies were conducted by trained interviewers 2 to 3 months after the death. Causes of death were ascertained by means of physician review. Initially the cause of death was assigned by two physicians independently. If the assigned cause of death for the two physicians was inconsistent it was reviewed by the third physician. If the cause of death assigned by the third physician did not match with either of the two; the three physicians sat for panel and assigned the cause of death.

Data on site of death and other individual level characteristics were taken from the verbal autopsy. Place of death was identified from the caregivers of the deceased as either home, hospital, clinic, health center, at holy water, or any other. For the purpose of this study, we considered health facility versus outside health facility deaths.

Age was categorized as: 15 to 34, 35 to 54, 55 to 74, and 75 and older. Marital status was defined as single, married, widowed and divorced/separated. Education was categorized as: no education, primary education, secondary education, above secondary and other education (mosque and church). Occupational status was classified as: House wife, retired, professional/clerical/managerial, manual work, sales and services and unemployed.

Cause of death was categorized based on Global Burden of Diseases classification category [18] as HIV/AIDS, tuberculosis, infectious& parasitic diseases, respiratory conditions, cardiovascular diseases, malignant neoplasm, digestive diseases, other non communicable, undetermined and injuries. Since the frequencies of some of the non communicable diseases were very low we added all of them as other non communicable diseases.

We compared differences in socio-demographic characteristics and place of death using the *x*^2^-test for selected variables. We modeled the probability of dying in a health facility in a binary logistic model as a function of each of the socio-demographic variables. This procedure allowed estimation of the strength of the association between each variable and the probability of health facility death, taking into account the possible confounding effects of the other variables in the model. The principal measure of association was the odds ratio (OR).

### Ethical clearance

Verbal Autopsy interview was conducted after obtaining verbal informed consent from the kin or caregiver of the deceased after explaining the purpose and the procedure of the study. Information sheet prepared in English and translated to local language had been provided. Permission for the study had been also obtained from local authorities. Protocol of the program (ethical clearance) was approved by Institutional Review Board (IRB) of Medical Faculty, Addis Ababa University, and at the national level ethical clearance was obtained from the Ethiopian Science and Technology Agency. Government and institutional officials, religious leaders at each level had been communicated officially.

## Result

**Figure 1:**
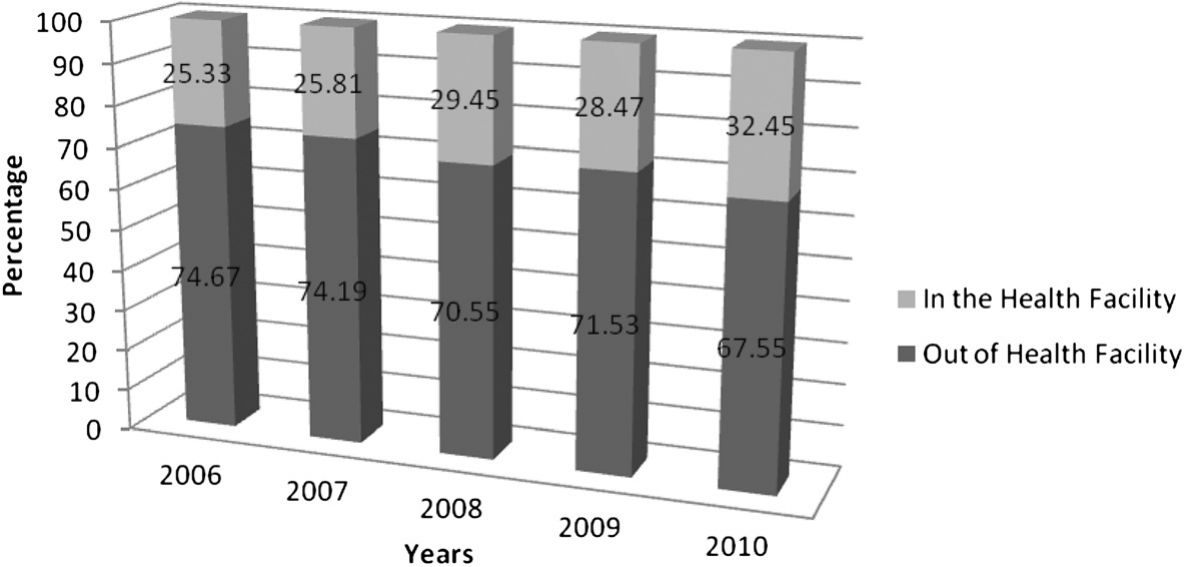
Place of death of patients who died in Addis Ababa from 2006 to 2010.

During the 5 years period there were 85,584 deaths registered from all cemetery sites of Addis Ababa. Verbal autopsy was administered for 2,397 (50.19%) females and 2,379 (49.81%) males during the five year study period. Only 28.7% died at health facilities, while the majority of them (71.3%) died out of health facilities. The proportion that died in health facilities increased during the 5 years period from 25.3% in 2006 to 32.5% in 2010 (Figure 1).

The percentage of deaths occurring in health facilities decreased as age increased; on the other hand the opposite occurred with respect to dying at out of health facilities (Table 1). The proportions of health facility deaths were almost similar for each gender.

**Table 1:** Bivariate analysis of place of death by sex, age, marital status, religion, ethnicity, educational status, occupational status and cause of death

**Variable**	**Out of HF (%)**	**HF (%)**	**Total (%)**	**P-value**
**Sex**				0.247
Male	1,677(49.28)	702(51.13)	2,379(49.81)	
Female	1,726(50.72)	671(48.87)	2,397(50.19)	
**Age**				0.000
15-34	570(16.75)	355(25.86)	925(19.37)	
35-54	794(23.33)	493(35.91)	1,287(26.95)	
55-74	994(29.21)	371(27.02)	1,365(28.58)	
>74	1,045(30.71)	154(11.22)	1,199(25.10)	
**Marital Status**				0.000
Single	645(18.95)	341(24.84)	986(20.64)	
Married	1,229(36.12)	633(46.10)	1,862(38.99)	
Widowed	1,126(33.09)	265(19.30)	1,391(29.12)	
Divorced/Separated	403(11.84)	134(9.76)	537(11.24)	
**Religion**				0.218
Orthodox	3,022(88.80)	1,202(87.55)	4,224(88.44)	
Others	381(11.20)	171(12.45)	552(11.56)	
**Ethnicity**				0.016
Amhara	1,739(51.10)	747(54.41)	2,486(52.05)	
Oromo	877(25.77)	295(21.49)	1,172(24.54)	
Gurage	416(12.22)	183(13.33)	599(12.54)	
Others	371(10.90)	148(10.78)	519(10.87)	
**Educational Status**				0.000
No education	1,582(46.49)	314(22.87)	1,896(39.70)	
Primary Education	781(22.95)	369(26.88)	1,150(24.08)	
Secondary Education	561(16.49)	395(28.77)	956(20.02)	
Above Secondary	196(5.76)	187(13.62)	383(8.02)	
Other Education	283(8.32)	108(7.87)	391(8.19)	
**Occupational Status**				0.000
House wife	1,134(33.32)	332(24.18)	1,466(30.70)	
Retired	548(16.10)	144(10.49)	692(14.49)	
Professional/Clerical/Managerial	140(4.11)	134(9.76)	274(5.74)	
Manual work	890(26.15)	413(30.08)	1,303(27.28)	
Sales and Services	332(9.76)	207(15.08)	539(11.29)	
Unemployed	359(10.55)	143(10.42)	502(10.51)	
**Cause of Death**				0.000
HIV/AIDS	422 (12.40)	247(17.99)	669(14.01)	
Cardiovascular diseases	718(21.10)	306(22.29)	1,024(21.44)	
Digestive diseases	187(5.50)	114(8.30)	301(6.30)	
Infectious and parasitic diseases	401(11.78)	113(8.23)	514(10.76)	
Injuries	237(6.96)	68(4.95)	305(6.39)	
Malignant neoplasm	361(10.61)	111(8.08)	472(9.88)	
Other non communicable disease	298(8.76)	154(11.22)	452(9.46)	
Respiratory conditions	145(4.26)	60(4.37)	205(4.29)	
Tuberculosis	270(7.93)	148(10.78)	418(8.75)	
Undetermined	364(10.70)	52(3.79)	416(8.71)	

The proportions of place of deaths were different with respect to marital status of the deceased. Married and single decedents showed the greatest proportion of health facility death. Decedents with no any education had lower proportions of deaths at health facilities than decedents of educated. About one in every four (26.88%) in health facility decedents had primary education compared to 22.95% for out of health facility decedents and 22.87% of in health facility deaths had no any education. The highest proportions of in health facility deaths were those who had secondary education (28.77%). Of those who died outside the health facilities, 16.49% of them had secondary education and 5.76% had above secondary education and 8.32% had other education (education from church or mosque). However, of those who died at health facilities 13.62% had above secondary education and 7.87% had informal education.

There was a proportionate difference in place of death among deceased by occupation. Among all who died at health facilities the proportion of professionals, managerial or clerical decedents was 9.76% this drops to 4.11% for those who had died out of health facilities. About one in four (24.18%) deceased at health facilities were house wives. Among all who had died out of health facilities 33.32% were house wives. 30.08% of deceased who had died at health facilities were working manual work compared with 15.08% for those who were working sales and services. The proportion of retired decedents for health facility deaths was only 10.49% but the proportion was 16.10% for out of health facility deaths. Of those who were unemployed, the proportions of decedents were equivalently split between outside health facility and at health facility (10.55% and 10.42% respectively).

Among deaths that occurred at health facilities, 17.99% were died of HIV/AIDS, 22.29% cardiovascular diseases, 8.30% digestive diseases, 8.23% infectious and parasitic diseases, 4.95% injuries, 8.08% malignant neoplasm, 11.22% died of other non communicable diseases, 4.37% respiratory conditions, 10.78% tuberculosis and 3.79% died of undetermined cases.

Table 2 shows odds of dying at health facilities compared to odds of dying out of health facilities and 95% confidence intervals from logistic regression analysis. The risk of health facility death versus out of health facility deaths decreased with age. The probability of health facility deaths for those 15–34 years age group was higher (adjusted OR: 2.73; 95% CI: 1.99, 3.73) compared with those >74 years of age. The probability was also higher for the 35–54 years of age (adjusted OR: 2.44; 95% CI: 1.87, 3.18); the probability was 1.88 times higher for the 55–74 years age group (95% CI: 1.51, 2.35) compared with ages >74 years.

**Table 2:** Crude and adjusted odd ratios of place of death

**Variable**	**Crude odd ratio**			**Adjusted odd ratio**		
	**Odd ratio**	**P-value**	**Confidence interval**	**Odd ratio**	**p-value**	**Confidence interval**
**Sex**						
Male						
Female	0.93	0.247	[0.82, 1.05]	1.27	0.024	[1.05, 1.54]
**Age Group**						
15-34	4.23	0.000	[3.41, 5.24]	2.73	0.000	[1.99, 3.73]
35-54	4.21	0.000	[3.44, 5.16]	2.44	0.000	[1.87, 3.18]
55-74	2.53	0.000	[2.06, 3.12]	1.88	0.000	[1.51, 2.35]
>74(RC)						
**Marital Status**						
Married(RC)						
Dissolved (Separated/divorced)	0.65	0.000	[0.52, 0.80]	0.68	0.001	[0.53, 0.86]
Single	1.03	0.753	[0.87, 1.21]	0.68	0.000	[0.56, 0.84]
Widowed	0.46	0.000	[0.39, 0.54]	0.70	0.000	[0.57, 0.85]
**Religion**						
Orthodox (RC)						
Others	1.13	0.218	[0.93, 1.37]	1.06	0.614	[0.85, 1.32]
**Ethnicity**						
Amhara (RC)						
Gurage	1.02	0.810	[0.84, 1.24]	1.03	0.797	[0.82, 1.29]
Oromo	0.78	0.002	[0.67, 0.92]	0.78	0.005	[0.66, 0.93]
Others	0.93	0.488	[0.75, 1.14]	0.95	0.631	[0.76, 1.18]
**Educational Status**						
No education (RC)						
Primary Education	2.38	0.000	[2.00, 2.83]	1.80	0.000	[1.48, 2.20]
Secondary Education	3.55	0.000	[2.97, 4.23]	2.62	0.000	[2.09, 3.29]
Above Secondary Education	4.81	0.000	[3.80, 6.07]	3.59	0.000	[2.68, 4.80]
Other (Informal Education)	1.92	0.000	[1.49, 2.48]	1.74	0.000	[1.33, 2.29]
**Occupational Status**						
Unemployed(RC)						
House wife	0.73	0.008	[0.58, 0.92]	1.14	0.397	[0.85, 1.52]
Retired	0.66	0.002	[0.51, 0.86]	0.90	0.502	[0.66, 1.23]
Professional/Clerical/Managerial	2.40	0.000	[1.77, 3.26]	1.35	0.089	[0.96, 1.90]
Manual work	1.16	0.186	[0.93, 1.46]	1.05	0.682	[0.82, 1.34]
Sales and Services	1.57	0.001	[1.21, 2.03]	1.29	0.072	[0.98, 1.71]
**Cause of Death**						
HIV/AIDS (RC)						
Cardiovascular diseases	0.24	0.000	[0.18, 0.34]	1.41	0.005	[1.11, 1.80]
Digestive diseases	0.73	0.003	[0.59, 0.89]	1.56	0.004	[1.15, 2.12]
Infectious& parasitic diseases	1.04	0.776	[0.79, 1.38]	0.90	0.469	[0.68, 1.20]
Injuries	0.48	0.000	[0.37, 0.63]	0.53	0.000	[0.38, 0.73]
Malignant neoplasm	0.49	0.000	[0.36, 0.67]	0.70	0.015	[0.53, 0.93]
Other non communicable diseases	0.53	0.000	[0.40, 0.68]	1.36	0.031	[1.03, 1.79]
Respiratory conditions	0.88	0.329	[0.69, 1.13]	1.39	0.081	[0.96, 2.01]
Tuberculosis	0.71	0.045	[0.50, 0.99]	1.21	0.173	[0.92, 1.58]
Undetermined	0.94	0.614	[0.73, 1.21]	0.47	0.000	[0.33, 0.66]

**Note**: The logistic regression analyses is adjusting for sex, age, marital status, religion, ethnicity, educational status and cause of death.

The chance of in health facility death was a little higher for females than males (adjusted OR: 1.27; 95% CI: 1.05, 1.54) while religion, occupation and ethnicity of the deceased had no any significance difference in place of death.

It was also shown that decedents who were single (not married) were 0.68 times less likely (95% CI: 0.56, 0.84) to die at health facilities as compared to those who were married. Moreover, it was found out that the widowed decedents were 0.70 times less likely (95% CI: 0.57, 0.85) to die at health facilities and the divorced or separated decedents were 0.68 times less likely (95% CI: 0.53, 0.86) to die at health facilities as compared with those who were married.

Educated people were more likely to die at health facilities than those who had no education. The probability of dying at health facilities was 1.80 times higher for those with primary education as compared with those with no any kind of education (95% CI: 1.48, 2.20). Decedents with secondary education had higher probability of dying at health facilities (adjusted OR: 2.62; 95% CI: 2.09, 3.29) as compared with those with no any education. The probability was also very high for those who had above secondary education (adjusted OR: 3.59; 95% CI: 2.68, 4.80) and it was 1.74 times higher for those who had other education (e.g. church, mosque) (95% CI: 1.33, 2.29).

As far as cause of death is concerned significance difference was observed between those who died of HIV/AIDS and some of other diseases. Decedents whose cause of death was cardiovascular diseases have odds of dying at health facilities between 1.1 and 1.8 times greater than those who died of HIV/AIDS. Furthermore, the risk of dying at health facilities was higher for digestive diseases (adjusted OR: 1.56; 95% CI: 1.15, 2.12) and other non communicable diseases (adjusted OR: 1.36; 95% CI: 1.03, 1.79). However, there was no significance difference observed between HIV/AIDS and respiratory conditions, infectious and parasitic diseases and tuberculosis. Decedents whose cause of death was malignant neoplasm were less likely to die at health facilities as compared with whose cause of death was HIV/AIDS (adjusted OR: 0.70; 95% CI: 0.53, 0 .93). Moreover, the probability of in health facility death was low for those decedents whose cause of death was injuries and undetermined.

## Discussion

The study was conducted in the urban setting of Ethiopia where most health facilities and medical environments are available relative to other parts of the country. In Addis Ababa, totally there are 43 (21% of the total hospitals in the country) hospitals of which 12 are registered public and 31 are registered private hospitals. In addition, there are 41 health centers (24 governmental and 7 private) with 141 beds and 551 private clinics (109 special, 169 higher, 146 medium and 127 lower). Nearly 43% of the total medical doctors in the country are serving in these health facilities [19]. However, we found that only 28.7% of the deceased died at health facilities while majority of them (71.3%) died out of health facilities. This result is very close to previous report 25% for the year 2003 [11] and higher than 19% for 2001 [20].

Over the years, there has been a trend towards the dying process moving from non health facilities to health facilities. This is evidenced by previous reviews which demonstrate increasing rates of medical facility death, and a reduction in non medical facility death [11,20].

Before considering the implication of this finding, some limitations have to be considered. A more important limitation is that we did not take into account all relevant patient information, which would have allowed us to control for all possible confounders. For example, since we did not have access to individual income data, we were unable to estimate the effect of personal or family income on place of death.

The study has identified some factors influencing the place of death in Addis Ababa, Ethiopia. In our study, both the descriptive and multivariate analyses showed that age of the deceased was strongly associated with place of death; as age of the deceased increased the probability of dying in the health facilities decreased. This finding is in line with some other studies that show place of death of the decedents has profoundly affected by age [11,21,22]. This may be an indication that younger people have more accessibility and knowledge of using health facilities as they have a better socioeconomic status to afford than older people [23].

In the descriptive analysis there were no differences in the proportion of place of deaths between males and females. However, there was a difference in the multivariate analysis between males and females. Females were more likely to die in a health facility than males is consistent with some studies [14,15] but not with others [21,22]. The possible explanation can be, when females are caregiver the death of the patient at home is more likely than when males are caregiver. Since the primary caregiver must be able to provide home care for the terminally ill if they are to die at home. That is, the traditional caring role of women is relatively better and common than males, that males may be less able or less willing to provide care at home [14,24]. Our result may strengthen also the fact that, in Sub-Saharan Africa, mostly women take on the role of caring family members during illness with other domestic activities at their home [25].

In this study, we found that being married was associated with an increased likelihood of health facility death. Independent of potential confounding factors, married persons died more frequently in health facilities compared with single, widowed and dissolved (separated or divorced). This finding is consistent with results indicating that married persons are more likely to die at health facilities than others [21,26] and inconsistence with others [15,27,28]. In the developed world individual’s place of death is a function of both preference and access. Being married in the developed countries is associated with dying at home, this is true because having social support at home facilitates being taken care of at home [29]. However, our result should not be explained in the context of developed countries. This opposite finding is mainly due to the different health care systems, availability and accessibility of services. Those decedents who were unmarried (single, divorced/separated and widowed) might not get the access as compared with married though they preferred to get care in health facilities. In resource limited areas like the case of Ethiopia, having spouse increases access to health facilities [23]. In societies like Ethiopia where family ties are very strong; besides personal experience and interest, family attitudes, beliefs and norms can provide great role for decision making during illness [30]. It is very clear that family members are crucial in taking the patient to medical facility, covering medical costs, providing physical and emotional care. One of the objectives of marriage is for mutual support; therefore if a married person is ill his/her partner can take him/her to the health facility and cover all the necessary costs during medication.

There was no significant difference between where orthodox persons die compared with other religions. Moreover, we did not find significant differences in place of death with regard to ethnicity. This result could be interpreted within the context of the characteristics of the study area. The most possible explanation would be that in urban areas the effect of religion and ethnicity on health service seeking behavior of individuals is minimal.

Not surprisingly, educational status influenced location of death. Educational statuses of the decedents were observed to affect site of death in that the percent of deaths with certain education was positively associated with dying in health facilities. After adjusting for other factors, higher educational status was more likely to favor death at health facilities. Again, this may reflect a greater knowledge about health facility, plus access to health services. It is clear that educated people has better information on health matters and hence use health facilities more frequently as compared with others [31]. Therefore, the probability that people visiting a health facility for the condition that leads them to death would be greater for patients with higher educational attainment. Moreover, it might be an indication that educated people would be more likely to have access and use health services than their counterparts. The other plausible explanation can be from the point of view of beliefs and norms of the society in which People who are less educated may visit holy water and traditional healers more frequently as compared with educated people. It is common that those who visited holy water in their final illness are less likely to have died in medical facility [11].

Another important factor affecting place of death is the underlying cause of death. In this study, patients with cardiovascular diseases, digestive diseases and other non communicable diseases are most likely to die at health facilities as compared with HIV/AID patients. This result may reflect a decrease in the use or access of health facilities by persons with AIDS in general as compared with persons with other diseases. The finding may also an indication that in response to the high prevalence of HIV/AIDS epidemic in Sub-Saharan Africa community home based care has become common [3,25]. Moreover, this may also reflect that people with HIV/AIDS receive home based care by different institutions like traditional burial societies which are common in Ethiopia [32].

Deaths from cancer were less likely to die at health facilities compared with HIV/AIDS deaths. One possible explanation for this can be communicable and infectious diseases like HIV/AIDS have got more attention as compared with non communicable diseases like cancer. However, non communicable diseases in the urban setting of developing countries like Addis Ababa are becoming dominant [33]. The other explanation could be, as the treatment of cancer patients is not that much developed in developing countries like Ethiopia [33,34]; therefore cancer patients may feel safe at home surrounded by their families and prefer to care at home by their family and think that it is convenient for those who want to visit them [10].

As expected, deaths from injury were less frequently died in health facilities. The most possible explanations for the higher incidence of deaths out of health facility for deaths due to injury might be most injury deaths happened before getting help in health facilities.

Place of end of life care provision is preceded by a decision making process that is further governed by individual and/or household behavior, community norms and expectations as well as provider related characteristics and behavior [25]. The low proportion of deaths at medical facilities in our finding may not be only due to access to health facilities. Apart from access to health facilities the norms, attitudes and beliefs of the society may have great influence where a person dies. Ethiopia is a country where cremation is not practiced and burials are conducted at dedicated religious and municipal burial sites by families of the deceased [35]. Therefore, if the patient is critically ill and with bleak prognosis the family members may prefer the patient to be discharged from medical facilities to reduce the cost and to get other religious activities for the dead body at home before burial. This is true because in most cases of the African context burials are given more emphasis by many people than the sickness. The other possibility is use of alternative traditional and religious means of healing. Although the health services are expanding in the last decade, many people in Ethiopia still use other means of healing. These means include using traditional healers who use various herbs and leaves and going to religious or worship places to get various interventions such as prayers, holy water for drinking and bathing, and anointing oils. This is common in both urban and rural settings [36].

## Conclusion

Having all the limitations stated, we believed that the study showed very important findings regarding place of death in Addis Ababa. Both demographic and social factors determine where adults will die in Addis Ababa, Ethiopia. The majority of people in Addis Ababa died out of health facilities, this can be an indication that most deceased were not visiting medical facilities for the conditions that lead them to death. Age, gender, educational status and marital status were predictors of the place of death but the educational status and age of the deceased seemed to be key predictors. The current health policy of Ethiopia is focusing more on communicable diseases. The health system should also give special attention to the emerging non communicable diseases like cancer for effective treatment of patients. Further research need to explain the effects of health system on place of death.

## Abbreviations

AAMSP: Addis Ababa Mortality Surveillance Program; CI: Confidence Interval; GBD: Global Burden of Diseases; OR: Odd Ratio; RC: Reference Category; VA: Verbal Autopsy; FDREMOH: Federal Democratic Republic of Ethiopia Ministry of Health; WHO: World Health Organization.

## Competing interests

We declare that we have no competing interests in connection with this paper.

## Authors’ contribution

**AA** has made substantial contributions to conception and design, analysis and interpretation of data, draft the manuscript and revising it critically. **TA** has made substantial contributions to conception and design, drafting the manuscript and revising it critically. **AM** has made substantial contributions to conception and design, drafting the manuscript and revising it critically. All authors read and approved the final manuscript.

## Authors’ information

**Aderaw Anteneh** is a Demographer/Researcher in Addis Ababa Mortality Surveillance Program, College of Health Sciences, Addis Ababa University. He has earned his Masters Degree in Demography/Population Studies from Addis Ababa University, Addis Ababa, Ethiopia.

**Tekebash Araya** is Addis Ababa Mortality Surveillance Program Manager, College of Health Sciences, Addis Ababa University. She has earned her PhD and MSc in Public Health, from School of Public Health, Addis Ababa University, Addis Ababa, Ethiopia.

**Awoke Misganaw** is a public health researcher in Addis Ababa Mortality Surveillance Program, College of Health Sciences, Addis Ababa University. He is a PhD candidate in public health, in the School of Public Health, Addis Ababa University. He has earned his Masters Degree in Public Health from School of Public Health, Addis Ababa University, Addis Ababa, Ethiopia.

## Pre-publication history

The pre-publication history for this paper can be accessed here:

http://www.biomedcentral.com/1472-684X/12/14/prepub
